# Bridging the Gap Between Environmental Adversity and Neuropsychiatric Disorders: The Role of Transposable Elements

**DOI:** 10.3389/fgene.2022.813510

**Published:** 2022-05-25

**Authors:** Holly DeRosa, Troy Richter, Cooper Wilkinson, Richard G. Hunter

**Affiliations:** Psychology Department, Developmental Brain Sciences Program, College of Liberal Arts, University of Massachusetts Boston, Boston, MA, United States

**Keywords:** glucocorticoids, retrotransposon, early life stress, epigenetics, schizophrenia, autism (ASD), PTSD - posttraumatic stress disorder, mood disorder

## Abstract

Long regarded as “junk DNA,” transposable elements (TEs) have recently garnered much attention for their role in promoting genetic diversity and plasticity. While many processes involved in mammalian development require TE activity, deleterious TE insertions are a hallmark of several psychiatric disorders. Moreover, stressful events including exposure to gestational infection and trauma, are major risk factors for developing psychiatric illnesses. Here, we will provide evidence demonstrating the intersection of stressful events, atypical TE expression, and their epigenetic regulation, which may explain how neuropsychiatric phenotypes manifest. In this way, TEs may be the “bridge” between environmental perturbations and psychopathology.

## Transposable Elements: An Overview

Transposable elements (TEs) are “selfish” sequences of DNA that replicate themselves across the genome. Barbara McClintock published the first account of TEs in 1950 after observing consistent breakages at certain loci in maize which had the ability to “transpose” and insert themselves into different positions along the genome. Interestingly, the loci also had a regulatory effect on the genes surrounding the new position; the expression of the adjacent genes was often lowered or even eliminated after these insertions. These were termed “mutable loci” or “controlling elements” are now classified as DNA transposons, which are one of the two major types of TEs. DNA transposons excise themselves from their position in the genome and mobilize to a different position via a “cut and paste” mechanism (McClintock, 1950; Muñoz-López and García-Pérez, 2010; Fischer and Suttle, 2011; Kalendar et al., 2011). The other class of TEs, retrotransposons, are more active in the brain than in any other human tissue (Reilly et al., 2013; Hunter, 2020). These elements mobilize around the genome via a “copy and paste” mechanism duplicating in the genome through its transcription into RNA, reverse transcription into cDNA, and insertion of the new DNA intermediate (Kalendar et al., 2010; Alzohairy et al., 2014). While all TEs constitute at least 45% of the human genome (Lander et al., 2001) DNA transposons are not active in humans (Pace and Feschotte, 2007), and will not be discussed in this review.

Retrotransposons can be further classified as Long Interspersed Nuclear Elements (LINEs), Short Interspersed Nuclear Elements (SINEs), and Human Endogenous Retroviruses (HERVs). HERVs are the remnants of ancient exogenous retroviral infections and make up roughly 8% of the human genome (Bock and Stoye, 2000; International Human Genome Mapping Consortium, 2001). HERVs have a basic genetic structure of about 9.5 kb in length, 2 long terminal repeats (LTRs), and four essential viral genes: gag, pro, pol, and env (Shin et al., 2013). In this way, HERV structure resembles that of modern endogenous retroviruses such as HIV (Yang et al., 1999). Most HERVs have mostly been silenced by mutations and deletions accumulated over millions of years, preventing full length transcription (Lander et al., 2001).

Some families of HERVs however, remain transcriptionally active in the human genome and may be involved in cellular function and disease susceptibility (Boller et al., 1993; Andersson et al., 2002) which will be discussed in later sections.

Despite McClintock’s discovery, TEs were long thought of as “junk” DNA (Orgel and Crick, 1980). However, TEs can act as *cis-*regulatory elements, such as transcription factor binding sites, that serve as enhancers and promoters ultimately affecting the transcription of eukaryotic genes (van de Lagemaat et al., 2003; Trizzino et al., 2017; Pontis et al., 2019). In addition to behaving as *cis*-regulatory elements, TE insertions can also promote alternative splicing or exonization which leads to the changing or prevention of transcription (Mersch et al., 2007; Abascal et al., 2015). We have argued that TE’s represent a substantial source of regulatory non-coding RNA, permitting greater adaptive capacity both to individual organisms and to populations as the evolve (Hunter et al., 2013; Hunter et al., 2015; Hunter, 2020). This adaptive capacity can go wrong of course, contributing to risk for a number of neurological and psychiatric disorders (See Table 1; Reilly et al., 2013; Daskalakis et al., 2018).

**TABLE 1:** Summary of TE effects organized by disorder.

Disorder	Authors	Model	Role of TEs
Schizophrenia	Basil et al. (2014)	Mice	↓ methylation of LINE-1 and Mecp2 in hypothalamus after MIA
	Bundo et al. (2014)	Human, Primate, Mice	↑ LINE-1 in prefrontal cortex of humans with schizophrenia, ↑ LINE-1 in macaque and mouse brain after MIA
	Guffanti et al. (2016)	Human	Non-reference TE insertions associated with pathogenesis of schizophrenia
	Guffanti et al. (2018)	Human, Primate, Rat	Differential expression of TEs in the dorsolateral prefrontal cortex of patients with schizophrenia
	Huang et al. (2006)	Human	↑ HERV pol expression in blood of patients with recent-onset schizophrenia
	Huang et al. (2011)	Human	↑ BDNF by HERV-W env
	Johansson et al. (2020)	Rat	HERV-W env interferes with glutamatergic synapse development in the hippocampus
	Karlsson et al. (2001)	Human	↑ HERV-W RNA homologs in frontal cortices of patients with schizophrenia
	Karlsson et al. (2004)	Human	↑ HERV-W gag sequences in plasma of patients with recent-onset schizophrenia
	Melbourne et al. (2018)	Human	↑ HERV-W env and gag after pro-inflammatory treatment in monocytes
	Nellåker et al. (2006)	Human	↑ HERV-W elements after viral infection
	Núñez Estevez et al. (2020)	Rat	↑ Mecp2 in placenta, ↓ Mecp2 in hypothalamus of females after MIA
	Page et al. (2021)	Primate	↓ TE regulators PIWIL2 and MGARP in DLPFC and ACC after MIA
	Tamouza et al. (2021)	Human	↑ HERV-W env in serum of patients with schizophrenia
	Yolken et al. (2000)	Human	↑ HERV-W expression in frontal cortex of patients with schizophrenia
ASD	Balestrieri et al. (2012)	Human	↑ HERV-H and ↓ HERV-W expression in peripheral blood mononuclear cells (PBMCs) of patients with ASD
	Balestrieri et al. (2016)	Human	↑ HERV-H, ↓ HERV-W, and HERV-K expression in PBMCs of patients with ASD
	Casanova et al. (2019)	Human	↑ TE number/density associated with ASD-related genes
	Cipriani et al. (2018)	Mice	↑ ERV & proinflammatory cytokines in embryo, blood, and brain of BTBR mice
	Matsumura et al. (2020)	Mice	POGZ mutation results in ASD-like phenotype
	Muotri et al. (2010)	Mice	↑ neuronal LINE-1 retrotransposition resulting from epigenetic MeCP2 mutations
	Shpyleva et al. (2017)	Human	↑ LINE-1 and ↓ H3K9me3 expression in cerebellum of individuals with ASD
	Wen et al. (2017)	Human	MeCP2 mutations associated with ASD
	Williams et al. (2013)	Human	↑ TE association with ASD-related genes
PTSD	Bartlett et al. (2021)	Rat	↓ H3K9me3 at B2 SINE loci leads to ↑ B2 SINE mobilization in response to acute corticosterone injection
	Cecco et al. (2019)	Mice	↑ inflammatory type-I interferon response as a result of ↑ LINE-1 expression due to senescence
	Elenkov and Chrousos (1999)	Human	↑ type 1 cytokine and ↓ type 2 cytokine secretion by glucocorticoids
	Hunter et al. (2012)	Rat	↑ H3K9me3 levels at ERV/LTR TE loci in hippocampus after stress
	Jones et al. (2015)	Rat	Administration of IL-1 antagonist inhibited development of stress-enhanced fear learning
	Lambert et al. (2020)	Rat	B2 SINE RNA levels in rat hippocampus correlate with stress coping strategies in a sex-specific manner
	Liu et al. (2016)	Rat	↑ malondialdehyde, IL-6, NOX2, & 4-hydroxynonenal after single prolonged stress
	Ponomarev et al. (2010)	Rat	↑ LINE-1 expression in amygdala after stress-induced learning
	Rusiecki et al. (2012)	Human	Individuals with PTSD had hypermethylated Alu before traumatic event and hypomethylated LINE-1 after
	Shintani et al. (1995)	Rat	↑ proinflammatory marker cytokine interleukin-1 after acute stress
	Thomas et al. (2017)	Human	↑ LINE-1 expression can lead to neuroinflammation
Mood Disorders	Perron et al. (2012)	Human	↑ HERV env present in peripheral blood of patients with BPD
	Tamouza et al. (2021)	Human	↑ HERV-W env present in serum of patients with BPD
	Weis et al. (2007)	Human	↓ HERV-W gag expression in patients with MDD and BPD

LINEs and SINEs can be distinguished from HERVs based on their lack of LTRs. LINEs in particular are the most prevalent in humans and activity of LINE-1 constitutes most of the retrotransposition in the human genome (Beck et al., 2011). SINEs include the primate derivative Alu, rodent specific B1 and B2 elements, as well as a conglomerate of SINE-R VNTR and Alu elements referred to as SINE-V A (SVA; Lapp and Hunter, 2019). Deleterious insertions of LINEs, SINEs, and SVAs have been linked to a variety of neurological and psychiatric diseases (Beauregard et al., 2008; Taniguchi-Ikeda et al., 2011; Guffanti et al., 2018). Thus, TEs must be managed in order to prevent the detrimental outcomes that can follow ungovernable TE mobilization. TEs are mostly silenced in the somatic genome by epigenetic regulations that prevent transcription. These regulations include DNA methylation and histone modifications (e.g., H3K9me3 and H3K7me3) which involve adding methyl groups to chromatin in order to make DNA less accessible for transcription (Hunter et al., 2012). TEs can also be suppressed through the process of RNA interference (RNAi). Small Interfering RNA (siRNA) is a form of non-coding, double-stranded RNA that degrades specific RNAs, often the RNA intermediates of retrotransposons (Tabara et al., 1999; Buchon and Vaury, 2006). And yet, despite these mechanisms, TEs insertions can still break through these biological defenses and negatively impact hosts. The epigenetic regulation of TEs can also be impacted by a number of environmental factors, highlighting one mechanism by which an organism’s environment directly impacts its DNA and even that of its progeny (Peschke et al., 1987; Lapp and Hunter, 2016). Interestingly, it has been hypothesized that epigenetic regulation evolved as a method of TE suppression (Choi and Lee, 2020).

### Roles of TE in Development and Neurodevelopmental Disease

TEs are necessary for several aspects of mammalian development, especially in the CNS. For example, TEs drive chromatin rearrangement within the 2-cell stage mouse embryo (Kruse et al., 2019), and LINE-1 RNA in particular promotes the self-renewal of embryonic stem cells (Percharde et al., 2018). Krüppel-associated box containing zinc finger proteins (KZFPs), which recognize TEs in a sequencespecific manner and form a protein complex (KAP-1) in embryonic stem cells (Matsui et al., 2010; Yang et al., 2017), drive embryonic genome activation through the suppression of SVA, HERV-K and HERV-W (Pontis et al., 2019). Furthermore, HERV-W env protein (otherwise called ERVWE1), encodes for the fusogenic protein syncytin-1 (Blond et al., 2000), which is expressed in high levels during human placental development mediating the fusion and proliferation of trophoblast cells (Kim et al., 1999; Mi et al., 2000; Frendo et al., 2003). HERV-W env has also been shown to regulate calcium influx via transient receptor potential cation channel subfamily C member 3 (TRPC3) which modulates the expression of the DISC1 gene (Chen et al., 2019). This process is important to developmental processes including neurogenesis, migration, and synaptogenesis (Brandon et al., 2009).

Early development is a time of heightened plasticity and TE activity can be disrupted by environmental perturbations leading to permanent physiological and behavioral changes. Stress-associated alterations in TE expression and activity are often studied using the maternal immune activation model (MIA). This model involves injecting a viral mimetic into pregnant animals to elicit an immune response and is used to assess offspring outcomes following exposure to gestational infection (Brown et al., 2009; Arsenault et al., 2014; Estes and McAllister, 2016; Kentner et al., 2018). MIA is a major risk factor for developing psychiatric disorders, mainly schizophrenia and autism spectrum disorders (ASD) in humans (Brown et al., 2004; Zerbo et al., 2013), respectively. Some animal models have also been able to link MIA with other disorders such as depression and anxiety (Estes and McAllister, 2016; Meyer, 2019), although more epidemiological work is needed in this field to discern the role of MIA these pathologies. The biological mechanisms that promote the increased risk between MIA and disorders like schizophrenia and ASD are not clear but may be a result of altered TE activity following exposure to a viral infection. For example, influenza A can increase HERV-W family placental protein syncytin-1 expression and decrease H3K9me3 expression on precipitated chromatin of CCF-STTG1 cells (Li et al., 2014). This suggests that MIA-triggering infections may have the capacity to increase the transcription of placental genes directly though epigenetic modifications at HERV-W loci. This involvement of TE activity in MIA is further supported by *in vivo* models. For example, MIA was shown to modulate tRNA-derived small fragment tRNA halves, which actively inhibit ERVs, within the placenta and fetal brain of mice (Su et al., 2020). Although this study did not assess how this affected the postnatal brain, other studies utilizing MIA as models of schizophrenia and autism demonstrate the pervasive effects of MIA on TE activity throughout development and will be discussed further.

## Schizophrenia

Schizophrenia is a complex neurodevelopmental disorder that is characterized by the presence of symptoms like delusions, hallucinations, disorganized speech, and negative affect (James et al., 2018; NIMH, 2020). While the mechanistic underpinnings of schizophrenia can be genetically derived (He et al., 2021) another risk factor is early life adversity, where certain environmental stressors (e.g., physical or emotional abuse, maternal infection) contribute to permeant changes in brain physiology and behavior (Bennouna-Greene et al., 2011; Kraan, et al., 2015; Rokita et al., 2020; Matheson et al., 2013). Stress in the prenatal environment can increase one’s risk for developing psychiatric disorders including schizophrenia. For example, the probability of developing a schizophrenia spectrum disorder was significantly greater in progeny from mothers who reported greater levels of daily stress throughout their pregnancy, although this was only true for males (Fineberg et al., 2016). Compounded with the role of environmental experiences, genetic heterogeneity in schizophrenia (Lee et al., 2012; Schwab and Wildenauer, 2013) makes discerning the etiology of this disorder extremely challenging at the individual level. Given the role of TEs in the promotion of genetic diversity, teasing apart the individual differences in schizophrenia may be possible through targeting TE activity.

Much of the research surrounding TEs and schizophrenia illustrate a differential expression of HERVs. HERV-W in particular is elevated in the blood of subjects with recent-onset schizophrenia but not present in control individuals (Karlsson et al., 2004; Huang et al., 2006, 2011; Perron et al., 2008, 2012; Tamouza et al., 2021). There is also evidence supporting an intersection between inflammatory genes and HERV-W with schizophrenia. For example, the upregulation of inflammatory cytokines such as IL-6, TNF-α, and IFN-γ yields persistent subclinical inflammation and has been linked to schizophrenia (Miller et al., 2011; Chase et al., 2015). While preliminary, Melbourne et al. (2018) reported an association between long non-coding RNA (lncRNA) and HERV-W expression with IL-6 and IFN- γ mRNA expression in primary cells from humans with schizophrenia (Melbourne et al., 2018). This is supported by previous findings that there is altered HERV-W expression in diseases with an inflammatory component, like schizophrenia (Li and Karlsson, 2016). One possible explanation for this could be cytokine stimulation that increases transcription factor binding to HERV promoter elements which was demonstrated in HERV-K (Manghera et al., 2016). More research is needed to determine if inflammation is the cause of or consequence to HERV-W expression. Recent evidence points to infection from exogenous viruses like HSV-1 and influenza A/WSN/33 as activators for HERV-W expression (Nellåker et al., 2006; Huang et al., 2011). It has been proposed that this expression of endogenous viral sequences by exogenous viral infection is a protective defense mechanism employed by the cell to prevent spread of the virus, which raises interesting questions about the degree to which TE “self-interest” and host interests align (Ponferrada et al., 2003; Nellåker et al., 2006).

Recently, Tamouza et al. (2021) explored the presence of HERV-W in individuals with schizophrenia. They found ∼41% of patients with schizophrenia were positive for HERV-W env protein in serum, whereas 96% of controls were negative (Tamouza et al., 2021), and these findings replicated a previous study (Perron et al., 2008). More importantly, researchers found that HERV-W expression correlated with increased serum levels of inflammatory cytokines, further implicating the association between inflammation and HERV-W in schizophrenia. There are remarkably few studies with sufficient enough power to make a conclusion about HERV-W expression in human brain tissue. However, there are some that attempt to investigate this. One study investigated RNA expression in the frontal cortex from four postmortem brains from individuals with schizophrenia. HERV-W expression was significantly elevated compared to the six control tissues (Yolken et al., 2000). Additionally, in the frontal cortices of schizophrenic patients, there were differentially upregulated transcription of RNA homologous to HERV-W compared to control (Karlsson et al., 2001). Finally, another study used a microarray-based analysis of HERV transcriptional activity in human brains, the authors could not report any abnormal expression of HERV-W elements in patients of schizophrenia or bipolar disorder (Frank et al., 2005). Until more conclusive evidence is found for differential expression of HERV-W in human brain, preclinical-models will continue to be important in answering this question. In an effort to answer this, Johansson et al. (2020) demonstrated a link between atypical expression of HERV-W env in the developing hippocampus and future behavior alterations in rats. Specifically, the induction of hippocampal HERV-W env in on the day of birth using electroporation was associated with later social and working memory impairments (Johansson et al., 2020). While social and working memory impairments are seen in individuals with schizophrenia (Lee and Park, 2005; Forbes et al., 2009), these symptoms are not exclusive to schizophrenia.

In addition to HERVs, LINE-1 has also been frequently implicated in schizophrenia. For example, increased LINE-1 has repeatedly been demonstrated in the prefrontal cortex of subjects with schizophrenia compared to control subjects and these insertions have been shown to be localized to genes that govern (GO0045202; GO0030054; GO0045211; Bundo et al., 2014; Doyle et al., 2017). Furthermore, Guffanti et al. (2018) found 1,689 differently expressed TEs in the dorsolateral prefrontal cortex (DLPFC) of patients with schizophrenia, and this differential expression of LINE 1 in DLPFC, and in other regions, has also been demonstrated in animal models of schizophrenia. RNA-sequencing of primate brains revealed dysregulated expression of LINE-1 silencers *PIWIL2* and *MGARP* in the DLPFC and anterior cingulate cortex of primates exposed to MIA (Page et al., 2021). In another model of MIA, LINE-1 and a regulator of LINE-1 methyl-CpG-binding protein-2 *Mecp2* were shown to be differentially expressed in mice exposed to MIA. Specifically, LINE-1 and *Mecp2* were hypomethylated in the hypothalamus (Basil et al., 2014). Similar results were also observed in the striatum, but these did not reach levels of statistical significance (Basil et al., 2014). In rats, MIA was associated with increased expression of the transcriptional repressor *Mecp2* expression in the placenta, and this was associated with a reduction of hypothalamic *Mecp2* in female offspring on gestational day 15 (G15; Núñez Estevez et al., 2020). Other epigenetic markers that work to reduce retrotransposition, including DNA methyl transferases (DNMTs) 1 and 3a, and O-GlcNAcylation (OGT), were also reduced in the hypothalamus of MIA offspring at G15 (Núñez Estevez et al., 2020). Fitzgerald et al. (2021) has since replicated this reduced epigenetic TE repression in six day-old mouse hypothalamus following a maternal separation model of early life stress. In addition, this reduced hypothalamic methylation was associated with hyperactivity as demonstrated by the open field and elevated plus maze in adult mice (Fitzgerald et al., 2021). Despite not being a model of schizophrenia, together these studies suggest that both prenatal and neonatal perturbations can have lasting effects on offspring brain development and behavior possibly through epigenetic unmasking. Although neither of these studies assessed TE activity directly, the involvement of epigenetic markers warrants future investigations. Overall, while this evidence presents a clear involvement of TEs in both humans and in animal models of schizophrenia, there is a general dearth of literature that is dedicated to uncovering how these differentially expressed TEs contribute to schizophrenia pathogenesis. More basic research is needed to tie these large effects in TE expression to more complex outcomes like symptomology and behavior.

## Autism Spectrum Disorders

Autism Spectrum Disorders (ASD) refers to a wide spectrum of neurodevelopmental disorders that are primarily characterized by impairments in communication and social interaction (Lord et al., 2001; NIMH, 2018). Similar to schizophrenia, ASD is believed to arise from a combination of heritable and environmental factors (Koufaris and Sismani, 2015; Andersson et al., 2018).

Hundreds of gene have been implicated in ASD etiology (Rylaarsdam and Guemez Gamboa, 2019). One risk factor for ASD may be genomic instability due to increased TE activity, as evidenced by more frequent TE localization to genes associated with ASD compared to genes that are not associated with ASD (Williams et al., 2013; Casanova et al., 2019).

Mutations in *Mecp2* lead to the development of Rett syndrome (Amir et al., 1999), and have more recently been implicated in the etiology of ASD (Wen et al., 2017). A neuronal knockout of *Mecp2* has been shown to increase LINE-1 retrotransposition *in vitro* (Muotri et al., 2010). While complete *Mecp2* knockouts *in vivo* are unattainable due to lethality, the use of conditional *Mecp2* knockouts could be an efficacious option to explore the sufficiency of LINE-1 in models of Rett syndrome and ASD. *Mecp2* conditional knockouts are also used in establishing rescue models (Li & Pozzo-Miller, 2012), and the future targeting of LINE-1 in these rescue models could offer substantial insight into the mechanisms by which LINE-1 is involved in these specific neurodevelopmental disorders. In support of this, LINE-1 upregulation has been found in postmortem cerebellum in individuals (ages 4–39) with ASD, and this was in tandem to decreased expression of H3K9me3 at open reading frames (ORFs) 1 and 2 (Shpyleva et al., 2018). This weakened epigenetic repression may have contributed to the differences in LINE-1 expression, although more work is needed to delineate the details of the pathway proposed by this study. Nonetheless, Individuals with ASD also exhibit mutations in the gene that codes for Pogo transposable element with zinc finger domain (POGZ). One recent study found mutations in POGZ to impair cortical development which is also observed in patients with ASD (Garcia-Forn et al., 2020; Matsumura et al., 2020). Researchers also observed impaired prepulse inhibition and social behavior in POGZ mutant mice, which is compatible with an ASD-like phenotype (Matsumura et al., 2020).

In addition to LINE-1 and POGZ, upregulated HERV expression has also been found in the blood mononuclear cells in patients with ASD (Balestrieri et al., 2012; Balestrieri et al., 2016). In a follow-up investigation to address these findings, 2 animal models of ASD were used: BTBR T + tf/J inbred mice, which exhibit behavioral abnormalities congruent with ASD, and CD-1 outbred mice treated *in utero* exposure to the histone deacetylase (HDAC) inhibitor valproic acid (VPA). ASD model mice demonstrated an upregulation in ERV expression in whole brain from gestational day 10 to adulthood (Cipriani et al., 2018). Furthermore, altered ERV expression correlated with an upregulation in inflammatory cytokines, but only in the BTBR model, indicating the importance of model specificity (Cipriani et al., 2018).

## Stress and Neuropsychiatric Disease

While the early developmental environment is a sensitive time for TE activity, TEs are also highly active in the adult brain (Muotri et al., 2005), especially in response to stress (Hunter et al., 2009; Hunter et al., 2012). Here, we will highlight trauma- and stress-mediated TE activity and how it contributes to the development of PTSD and mood disorders in adulthood.

## PTSD

Posttraumatic stress disorder (PTSD) is a severe pathological condition that can develop after a major traumatic event (Muotri et al., 2005). While approximately half of all US adults will experience at least one traumatic event, most will not develop this disorder. Only around 7% actually develop PTSD (although rates could be higher in areas affected by natural disaster or warfare (Yehuda et al., 2015; Shahmiri Barzoki et al., 2021). Most of the research linking PTSD and transposable elements has focused on the role of LINEs, SINEs, ERV/LTRs and the epigenetic regulation of these elements utilizing both humans and animal models. In the rodent model, the molecular mechanisms of PTSD can be investigated using stressenhanced fear learning (SEFL) induced by repetitive foot shocks. In one particular study (Ponomarev et al., 2010), used SEFL to explore the transcriptional changes in the amygdala after these repetitive foot shocks. They found an upregulation of LINE-1 in the rat amygdala, supporting a potential role of this TE in stress-induced learning. One pioneering human study also found that LINE-1 to be hypomethylated in veterans with PTSD after deployment, while Alu was hypermethylated before deployment (Rusiecki et al., 2012). These results suggest that TEs can be both a protective or risk factor for getting PTSD after stressful events.

B2 SINE expression in the amygdala and hippocampus appears to be regulated by stress, where levels of B2 RNA have been shown to predict coping strategies in a sex specific fashion (Hunter et al., 2012; Lambert et al., 2020). Furthermore, glucocorticoids have been shown to promote B2 SINE mobilization *de novo* in the hippocampus and in cell lines (Bartlett et al., 2021), further underscoring the explicit interactions that occur between stress and the deep genome. These results support the notion that environmental stressors mediate TE activity through the promotion of interactions with the glucocorticoid receptor.

In addition to glucocorticoid receptor activation, several studies have shown that high levels of stress also promote the expression of pro-inflammatory markers such as cytokine interleukin (IL-1β) (Shintani et al., 1995; Miller et al., 2002).

Additionally, administration of an IL-1 antagonist prevented SEFL (Jones et al., 2015). Other pro-inflammatory markers exhibit altered expression following exposure to stress such as 4-hydroxynonenal, IL-6, malondialdehyde, and NOX2 (Elenkov and Chrousos, 1999; Jones et al., 2015; Liu et al., 2016). Upregulation of inflammatory cytokines is a common feature in a panoply of psychiatric diseases, including PTSD (Bam et al., 2016; Baumeister et al., 2016; Wang et al., 2019). Recently, it has been shown that a lack of LINE-1 repression can act as a driver for inflammation (Thomas et al., 2017) especially in aging cells (De Cecco et al., 2019).

Whether or not stress can mediate inflammation independently of LINE-1 however, remains to be elucidated. Nonetheless, this evidence suggests deleterious TE activity that intersects with region-specific glucocorticoid function and inflammation could be a risk factor for developing PTSD (Hori and Kim, 2019).

## Mood Disorders

Major depressive disorder (MDD) and bipolar disorder (BPD) are complex multigenic diseases that can arise from interactions between endogenous genetic risk factors, and exogenous risk factors such as childhood adversity and substance abuse (Anda et al., 2002; Hosang et al., 2017; Herzog and Schmahl, 2018; Misiak et al., 2018; Zhao et al., 2018). MDD has a lifetime risk of about 16% in the US population and is marked by episodes of depressed mood lasting for more than 2 weeks associated with other symptoms like depressed appetite, reduced energy, slow movements and sometimes suicidal thoughts (Murray and Lopez, 1997; Kessler et al., 2003; Mill and Petronis, 2007) (BPD) is a serious mood disorder that is marked by periods of both acute manic episodes and periods of acute major depressive episodes (Ghaemi, 2007). The prevalence of BPD ranges from 1–4% of the adult population (Tohen and Angst, 2002; Judd and Akiskal, 2003).

Decreased expression of human endogenous retroviral protein HERV-W gag is present in brain of patients with major depression and BPD (Weis et al., 2007), although this molecular mark is also present in patients with schizophrenia, making it not exclusive to mood disorders. It is worth noting in this context that genome wide association studies have shown substantial genomic overlap between neuropsychiatric disorders, and that many of the vulnerability loci reside in noncoding regions of the genome (Anttila et al., 2018). Additionally, HERV env sequences are associated with BPD as detected in the peripheral bloodof patients compared with healthy controls (Perron et al., 2012). Specifically, Tamouza et al. (2021) also found ∼28% of BPD patients were positive for HERV-W env protein in serum. Importantly, although HERV env was also found in peripheral blood of patients with schizophrenia, it was higher in patients with BPD, indicating some exclusivity to BPD (Perron et al., 2012).

Mood disorders are often interwoven with a dysregulated inflammatory response (Miller and Raison, 2016; Chistyakov et al., 2018; Jones et al., 2020). While HERV-W env has been shown to elicit the production of inflammatory cytokines including IL-6 and TNF-alpha, this was explored in models of multiple sclerosis *in vitro* (Perron et al., 2001; Rolland et al., 2005).

Additionally, one recent study found simulation of lymphocytes with a HERV-K env peptide significantly upregulated expression of IL-6 in patients with amyotrophic lateral sclerosis (ALS) but not in healthy controls (Arru et al., 2021). It has been hypothesized that cytokines may be similiarly elicited in the brain following aberrant HERV insertions within certain regions, especially hippocampus and amygdala, after exposure to stress or infection. These exogenous events can lead to disruption of the blood brain barrier and the subsequent development of psychiatric disease (Canli, 2019). As previously discussed, inflammation and the subsequent neurophysiological damage is also prevalent in disorders like schizophrenia and autism. Whether or not TE activity contributes to a unique inflammatory profile between these disorders still needs to be elucidated.

## Sex Differences

Almost every psychiatric disease exhibits a sex difference in either its prevalence or symptomology. For example, boys are at a higher risk for disorders of development, such as autism, ADHD, or early onset schizophrenia, whereas women are more commonly diagnosed with stress related disorders like depression or

PSTD (Bangasser and Valentino, 2014; McCarthy et al., 2017; McCarthy and Wright, 2017). There is a rapidly growing literature surrounding the critical involvement of TEs in male and female specific development (for review, see Dechaud et al., 2019). For example, sex differences in histone acetylation of estrogen receptor alpha and aromatase promoters by HDAC2 and HDAC4 are necessary during the critical period of sexual differentiation to properly masculinize the brain and behavior in rats (Matsuda et al., 2011). However, studies assessing the role of TEs in sex-specific disease susceptibility are almost absent. In one groundbreaking study, hippocampal B2 SINE RNA expression was increased in male rats and this correlated with sex-specific coping style in response to stress (Lambert et al., 2020). These results shed light on how TEs may mediate stress and susceptibility to stress related disorders in males and females distinctly. The future assessment of sex differences in TE expression and modulation in both healthy and clinical populations is warranted.

## Conclusion

Comprising almost half of the entire genome, TEs have garnered substantial interest given their involvement in psychiatric disease. Mounting evidence demonstrates interactions with the stress system, in both early development and adulthood, leads to deleterious TE activity that coincide with permanent changes in brain physiology, especially inflammation, leading to pathology. Inflammation may also be induced by the activation of cellular innate immunity by ectopic expression of TE RNAs in a number of tissues. Further, TEs contribute to somatic mosaicism, making them an appealing avenue to explore individual differences in both psychiatric diseases and in normal variation of cognitive and behavioral traits. However, substantial research needs to be done, as TEs have received a miniscule fraction of the research attention that protein coding elements of the genome have to date. If, as their evolutionary origins would suggest, TEs are acutely sensitive to host stress, then they represent a means by which the environment can program the genome across the lifespan, for both good and ill, a hypothesis that deserves a much deeper analysis.
